# Fighting Noise with Noise: A Stochastic Projective Quantum Eigensolver

**DOI:** 10.1021/acs.jctc.4c00295

**Published:** 2024-07-02

**Authors:** Maria-Andreea Filip

**Affiliations:** Yusuf Hamied Department of Chemistry, University of Cambridge, Cambridge CB2 1EW, U.K.

## Abstract

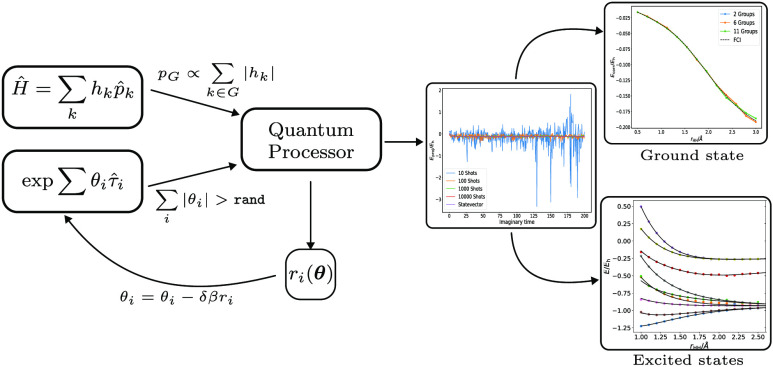

In the current noisy intermediate scale quantum era of quantum computation, available hardware is severely limited by both qubit count and noise levels, precluding the application of many current hybrid quantum-classical algorithms to nontrivial quantum chemistry problems. In this paper we propose applying some of the fundamental ideas of conventional Quantum Monte Carlo algorithms—stochastic sampling of both the wave function and the Hamiltonian—to quantum algorithms in order to significantly decrease quantum resource costs. In the context of an imaginary-time propagation based projective quantum eigensolver, we present a novel approach to estimating physical observables which can lead to an order of magnitude reduction in the required sampling of the quantum state to converge the ground state energy of a system relative to current state-of-the-art eigensolvers. The method can be equally applied to excited-state calculations and, combined with stochastic approximations of the system Hamiltonian, provides a promising near-term approach to Hamiltonian simulation for general chemistry on quantum devices.

## 1 Introduction

Electronic structure problems are hard for conventional computers, due to the exponential scaling of the Hilbert space with the physical size of the studied system. Quantum computers have been heralded as a solution to this problem,^1,2^ mainly due to their ability to use the quantum entanglement of their constituent qubits to encode even highly complex electronic wave functions in a linearly scaling qubit register. This promise has led to the development of a variety of quantum algorithms with applications in quantum chemistry, such as Hamiltonian simulation^3−6^ and Quantum Phase Estimation.^7,8^

However, while these techniques promise general solutions to many problems in quantum chemistry, they require many-qubit, fully fault-tolerant quantum computers to run. Currently we are in an era of Noisy Intermediate Scale Quantum (NISQ) hardware,^9^ which is characterized by moderate numbers (10–1000) of qubits prone to high error rates due to short coherence times and noisy gate implementations.^10^ While significant work is being carried out in the fields of quantum error correction^11−14^ and mitigation,^15−18^ the fully fault-tolerant quantum computer remains a thing of the future.

In order to make the most of currently available quantum resources, hybrid quantum-classical algorithms^19−25^ have been developed, which shift most of the computational overhead onto a classical processor, while requiring specific, highly efficient operations to be carried out by the quantum processor. The most well-known of these is the variational quantum eigensolver (VQE),^20^ in which some parametrized wave function is encoded on a quantum device and used to compute a cost function—usually the expectation value of a Hamiltonian operator. This is used in the classical optimization of the parameters. In quantum chemistry, this approach has been used to calculate ground-state energies of molecules and model systems.^20,26,27^

Since methods like VQE require estimating expectation values of quantum observables, despite making use of only moderately sized quantum circuits they require a large number of repeated quantum measurements to obtain sufficiently accurate estimators. This is due to the intrinsic noise associated with the measurement of a quantum observable. Significant work has gone into devising more efficient measurement schemes,^28−31^ which obtain lower variances for the same number of measurements.

In this paper, we approach the issue of decreasing quantum computational overhead of electronic structure problems by taking inspiration from conventional Quantum Monte Carlo (QMC) algorithms,^32−34^ which use noisy representations of the wave function and stochastic propagation techniques to obtain very high-accuracy results for challenging systems. In particular, we consider a projective quantum eigensolver (PQE)^35^ approach and propose an algorithm to estimate the energy *during* the propagation, which is found to require significantly fewer samples for the same accuracy as its conventional counterpart.

In a perfectly noiseless regime, the PQE algorithm converges smoothly to the ground state of a given Ansatz. However, in practice, it processes data obtained from finitely many samples of a distribution stored in a quantum device, which is intrinsically stochastic even when ignoring NISQ-era device noise. Achieving low enough uncertainty in this data to use it as if it were exact requires a large number of measurements. It is therefore compelling to employ instead an algorithm intended to operate on noisy measurements, such as QMC.

First, we establish a methodology to obtain accurate estimators for the ground state energy of a quantum system using relatively high-variance samples from an ongoing PQE propagation. We refer to this method as Monte Carlo PQE (MC-PQE). We then introduce additional stochastic approximations to further decrease quantum resource requirements. Stochastically rounding the wave function allows gates to be removed from the state preparation circuit, reducing its depth. The observables may also be estimated using a sampled subset of the Hamiltonian operator, lowering the number of independent measurements required. These techniques introduce additional noise into the algorithm, but we find this can be efficiently averaged out and, for a set of first row hydrides, show that the accuracy of the original algorithm can be recovered at significantly lower cost. Finally, we investigate the applicability of MC-PQE for excited states, using the folded-spectrum method,^20,36,81^ and find that, while convergence is somewhat more challenging, it can reduce the cost of an excited state calculation to that of the ground-state method. Given that the main drawback of the folded-spectrum method is its unfavorable scaling with system size, this is a very promising alternative.

In section 2 we present the underlying theory of quantum eigensolvers, conventional QMC methods, and the folded-spectrum approach which allows these algorithms to be expanded to excited state calculations. In section 3 we describe the quantum algorithm employed in this paper, together with circuit implementations and possible stochastic variations. Finally, in section 4 we apply these methods to a range of molecular systems and discuss their performance. We draw our final conclusions in section 5.

## 2 Background Theory

### 2.1 Variational Quantum Eigensolver

One of the principal NISQ algorithms for quantum chemistry is the variational quantum eigensolver (VQE).^20^ According to the variational principle, for a parametrized wave function Ψ(**θ**) that satisfies the boundary conditions of a problem with corresponding Hamiltonian *Ĥ*, the expectation value of the energy is greater than or equal to the ground state energy of the system *E*_0_,
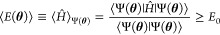
1Therefore, by minimizing ⟨*E*(**θ**)⟩ with respect to the set of parameters **θ**, one can obtain the best possible approximation of a given form to the ground state wave function. In VQE, the expectation value of the energy and any gradients used are computed by a quantum processor before being passed on to a classical optimization algorithm.

There are various considerations to be taken into account when choosing parametrized wave function Ansätze for VQE. Heuristic hardware efficient Ansätze (HEA)^37^ attempt to introduce many degrees of freedom while maintaining low circuit depths. However, optimization on the resulting landscapes is often difficult, as they are plagued by barren plateaus^38,39^—regions of parameter space in which the gradient vanishes exponentially with system size—that can prevent the optimization algorithm from converging to a true minimum. The propensity for a problem to display barren plateaus is dependent on a variety of system and algorithm properties^40^ and has been shown to be worse for more expressive Ansätze^41^ and higher degrees of entanglement.^42^ However, the intrinsic noise resulting from finite sampling of the quantum state can be helpful in improving optimization outcomes.^43^

Alternatively, one can use physically motivated Ansätze such as unitary coupled cluster (UCC),^44−47^ in which the wave function is expressed as
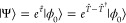
2where |ϕ_0_⟩ is typically the Hartree–Fock wave function,
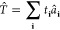
3
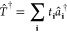
4**i** is an index running over all determinants in the Hilbert space and *â*_**i**_ and *â*_**i**_^†^ are Fermionic excitation and de-excitation operators, respectively, such that *â*_**i**_|ϕ_0_⟩ = ±|ϕ_**i**_⟩ and *â*_**i**_^†^|ϕ_**i**_⟩ = ±|ϕ_0_⟩, with signs derived from the anticommutation relations of creation and annihilation operators. The cluster amplitudes *t*_**i**_ are optimized to give an approximation of the ground state wave function. In principle, τ̂ can include up to all-electron (de)excitations, but it is common to truncate it to some low excitation order, most commonly including only up to two-electron operators, which gives the unitary coupled cluster singles and doubles (UCCSD) Ansatz. This type of Ansatz is generally found to be easier to optimize than HEAs but has its own challenges. In order to express the UCC operator in the set of gates available on most quantum computers, one generally has to perform a Trotter decomposition^48,49^ of the cluster operator,
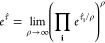
5where τ̂_**i**_ = *t*_**i**_(*â*_**i**_ – *â*_**i**_^†^) and we have rewritten the exponential in eq 2 as exp(*T̂* – *T̂*^†^) = exp(∑_**i**_ *t*_**i**_(*â*_**i**_ – *â*_**i**_^†^)) = exp(∑_**i**_ τ̂_**i**_). Usually, rather than working in the large ρ limit, ρ is taken to be unity, leading to a disentangled form of the UCC Ansatz. For the case of full UCC, this form has been found to be as expressive as the original algorithm in most circumstances, provided an appropriate operator ordering is used in the Trotter product.^50^

Because of this decomposition, UCC-based circuits often tend to be prohibitively deep for NISQ devices. Various alternative algorithms have been devised, that attempt to enforce some of the known physical properties of the wave function to generate easier to optimize energy functions, while maintaining a reduced circuit depth relative to UCC. Examples include symmetry preserving Ansätze,^51−54^ adaptive Ansätze,^25,35,55,56^ as well as methods like qubit coupled cluster (QCC)^57−59^ and Givens-rotation-based Ansätze,^60^ which do not employ Fermionic excitation operators.

### 2.2 Projective Quantum Eigensolver

As an alternative to variational algorithms, one can solve electronic structure problems using projective approaches. In classical settings, this technique is most commonly employed in solving the Coupled Cluster (CC) equations^61,62^ and Projector Monte Carlo (PMC) algorithms, which will be discussed in more detail in section 2.3.

Consider a trial wave function expressed as |Ψ⟩ = *Û*(**θ**)|ϕ_0_⟩, where |ϕ_0_⟩ is a simple reference wave function such as a Hartree–Fock (HF) determinant and *Û*(**θ**) is some parametrized unitary operator. If this were an exact ground-state solution to the Schrödinger equation, then
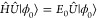
6and
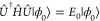
7Projecting eq 7 onto the reference wave function |ϕ_0_⟩ gives
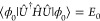
8while projecting onto the complete set of |ϕ_**i**_⟩ orthogonal to |ϕ_0_⟩ gives a set of quantities referred to as residuals
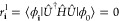
9where the superscript l denotes that these residuals are obtained from the linked formalism of the unitary coupled cluster equations.^63^

If *Û* has fewer than *N*_Hilbert_ – 1 parameters, where *N*_Hilbert_ is the number of states in the Hilbert space, eq 9 can only be enforced for a subset of determinants. Solving eqs 8 and 9 for this subset to obtain values of the parameters in *Û*(**θ**) generates an approximation to the true ground state. This method is known as the Projective Quantum Eigensolver^35^ algorithm.

If *Û* is a UCC wave operator and |ϕ_0_⟩ is a single Slater determinant, eqs 8 and 9 can be solved using a quasi-Newton iterative scheme such as^35^
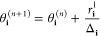
10If |ϕ_**i**_⟩ labels |ϕ_*ij*..._^*ab*...^⟩, with indices *i*, *j*, ... (*a*, *b*, ...) denoting orbitals occupied (unoccupied) in the reference determinant |ϕ_0_⟩, from (to) which electrons are excited, then the denominator Δ_**i**_ = *F*_*ii*_ + *F*_*jj*_ + ... – *F*_*aa*_ – *F*_*bb*_ – ..., where *F*_*pp*_ denotes diagonal elements of the Fock operator. If the HF determinant is used, then Δ_**i**_ = ϵ_*i*_ + ϵ_*j*_ + ... – ϵ_*a*_ – ϵ_*b*_ – ..., where ϵ_*i*_ are Hartree–Fock spin-orbital energies. The residuals *r*_**i**_^l^ can be computed using expectation values of equivalent cost as the overall energy, as either^35^
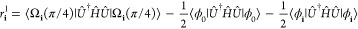
11or equivalently^64^
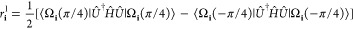
12where |Ω_**i**_(θ)⟩ = *e*^*θτ̂*_**i**_^|ϕ_0_⟩ = *e*^θ(*â*_**i**_ – *â*_**i**_^†^)^|ϕ_0_⟩ = cos θ|ϕ_0_⟩ ± sin θ|ϕ_**i**_⟩. The cost of PQE residuals is therefore comparable to that of VQE gradients computed using the parameter-shift rule, which also employs additional energy measurements to obtain the gradient elements.^65−68^

### 2.3 Quantum Monte Carlo

As mentioned above, one of the main uses of projective methods in conventional quantum chemistry is in PMC algorithms, which are based on the imaginary-time Schrödinger equation,
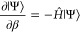
13where β is imaginary time. The lowest-energy solution to eq 13, |Ψ_0_⟩, is given, up to a normalization constant, by
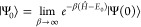
14where |Ψ(0)⟩ is some initial trial wave function such that ⟨Ψ_0_|Ψ(0)⟩ ≠ 0 and *E*_0_ is the lowest eigenvalue of *Ĥ*. The time-evolution operator can also undergo a Trotter expansion, giving
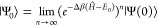
15where Δβ = β/*n*. For small Δβ, the exponential can be approximated by a linear expansion, leading to the form of the imaginary time propagator most commonly used in projector methods,
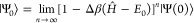
16Considering a single time-step Δβ, one can write
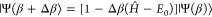
17which describes the stepwise imaginary time evolution of the trial wave function |Ψ⟩. As in PQE, this equation can be projected onto the various Slater determinants in the Hilbert space to give
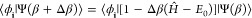
18

In the original Full Configuration Interaction Quantum Monte Carlo (FCIQMC) algorithm,^32^ the wave function is parametrized as a linear expansion in all determinants in the Hilbert space, |Ψ⟩ = ∑_**i**_ *c*_**i**_|ϕ_**i**_⟩. Eq 18 can then be rewritten as
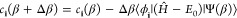
19or
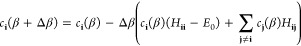
20where *H*_**ij**_ = ⟨ϕ_**i**_|*Ĥ*|ϕ_**j**_⟩. We note that this is equivalent to
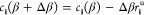
21where *r*_**i**_^u^ = ⟨ϕ_**i**_|(*Ĥ* – *E*_0_)|Ψ(β)⟩, with the superscript u now denoting an unlinked formalism.^63^

In FCIQMC, eq 20 is interpreted as governing the population dynamics of a set of signed particles (“walkers”, “psips”, or “excips”) living in the Hilbert space, such that on each determinant there exists a particle population such that configuration interaction (CI) coefficient, *c*_**i**_ on each determinant is proportional to the signed sum of this population. Rather than iterating these equations exactly, their terms are sampled stochastically and can be described by three processes:^32^

- **Spawning** of particles from determinant |ϕ_**j**_⟩ to |ϕ_**i**_⟩ corresponds to the off-diagonal action of the Hamiltonian
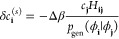
22where *p*_gen_(ϕ_**i**_|ϕ_**j**_) is the conditional probability of selecting ϕ_**i**_ as the target of a spawn given it originates from ϕ_**j**_, *k* is the index of the current attempt, and *s* denotes that this is a spawning event.
- **Death** or cloning from |ϕ_**i**_⟩ to itself corresponds to the diagonal action of the Hamiltonian
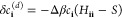
23where *d* denotes that this is now a death/cloning event.
- **Annihilation** ensures that the two equivalent oppositely signed versions of the wave function do not proliferate simultaneously. During the previous steps, multiple new walkers may be generated on the same determinant, sometimes with opposite signs. Annihilation corresponds to canceling out these contributions to leave a single-sign population on each determinant.

We note that in the death step the unknown true ground state energy *E*_0_ has been replaced by the shift *S*. This acts as a population control parameter. In standard QMC procedures, once the simulation has reached a target population, the shift can be used to keep it constant and eventually converges to a stochastic estimator of *E*_0_.^32^ This target population must exceed a minimum “plateau”, the value of which increases with system size and degree of correlation, but is normally smaller than the size of the Hilbert space.^32^ The projected energy
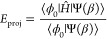
24is normally used as a second way to estimate *E*_0_.

By employing a stochastic representation of the wave function, algorithms like FCIQMC take advantage of the sparsity of the Hamiltonian, and are therefore able to tackle problems far beyond those tractable for conventional FCI solvers.^69^ This behavior can be further enhanced by developments such as the initiator approximation^70^ and the adaptive-shift method,^71,72^ which artificially stochastically increase the sparsity of the Hamiltonian.

Alternative projective Monte Carlo formulations have been developed using Coupled Cluster (CC)^33^ and UCC^34^ parametrizations of the wave function. These are based on the fact that for an exponential Ansatz, if a determinant |ϕ_**i**_⟩ is reachable by an excitation included in the cluster operator, then , where *t*_**i**_ are the cluster amplitudes. Since these methods are only valid in the regime in which the cluster amplitudes are small, a similar equation to eq 20 can be written down as
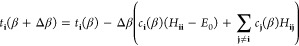
25Therefore, similar stochastic propagation techinques can be used as in the case of FCIQMC, however one only stores the cluster amplitudes *t*_**i**_. The corresponding CI coefficients appearing in eq 25 must also be estimated and this is done by stochastic sampling, leading to additional complexity in the selection routine, which is responsible for a significant portion of the computational cost of these algorithms.^73^

In FCIQMC it is easy to cycle through all determinants present in the wave function or sample them uniformly. In contrast, in Coupled Cluster Monte Carlo (CCMC) a doubly excited determinant |ϕ_**i**_⟩ = |ϕ_*ij*_^*ab*^⟩ will have a CI coefficient given by
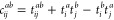
26where *t*_*i*_^*a*^, *t*_*ij*_^*ab*^ are the amplitudes of the corresponding single and double excitation operators *â*_*i*_^*a*^ and *â*_*ij*_^*ab*^ in the cluster operator *T̂*. Therefore, when selecting a determinant for spawning or death, one must consider both composite (e.g., *t*_*i*_^*a*^*t*_*j*_^*b*^) and noncomposite (*t*_*ij*_^*ab*^) contributions (“clusters”) to it. The standard way to do this in CCMC^33,73^ is to

1. Select a cluster size *s* with some probability *p*_*s*_;
2. (Optionally) select a particular type of cluster of size *s* by imposing constraints on the excitation levels of the individual excitors involved;^73^
3. Select *s* excitors *e* to form the cluster, each with probability *p*_*e*_ ∝ *t*_*e*_;
4. “Collapse” the excitors to determine the corresponding determinant (e.g., *â*_*i*_^*a*^*â*_*j*_^*b*^|ϕ_0_⟩ = ±|ϕ_*ij*_^*ab*^⟩).

This procedure leads to an overall probability of selecting a cluster corresponding to some determinant ϕ_**j**_, *p*_select_(ϕ_**j**_), and the spawning and death equations must be modified to account for this as
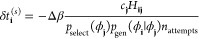
27
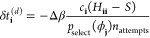
28where *n*_attempts_ is the number of attempted cluster selections. In the simplest implementation, this is chosen to be equal to the number of walkers in the system. A variety of selection algorithms have been developed for CCMC to better importance sample the underlying wave function.^73^ Nevertheless, this remains one of its main challenges, particularly when expanded to high truncation levels^74^ or multireference formulations.^75^

To further complicate matters, in unitary CCMC (UCCMC)^34^ the maximum cluster size is formally infinite, due to alternating excitation and de-excitation operators in the clusters. While results converge relatively quickly with maximum cluster size, the inclusion of large composite clusters leads to instabilities in the Monte Carlo propagation.

Finally, we note here that the applicability of this approach is not necessarily limited to chemically inspired wave function Ansätze, but should in principle be applicable to any wave function formalism in which a one-to-one correspondence between some states in the Hilbert space and the parameters can be constructed and where for such a state |ϕ_**i**_⟩, , where *N* is a multiplicative constant.

### 2.4 Folded-Spectrum

Methods like VQE, PQE, and standard PMC are all intended to find the ground state of a system of interest. The energy of some excited states can be found with these techniques by enforcing orthogonality between the state of interest and the ground state, for example by considering different symmetry sectors of the Hilbert space.^23^ However, obtaining general excited states is nontrivial.

Using the FCIQMC approach, it is possible to obtain multiple excited states simultaneously by instantaneously orthogonalizing the stochastic wave function representations of multiple independent FCIQMC calculations.^76^ A similar idea forms the basis for the Variational Quantum Deflation (VQD)^22^ algorithm, although this requires sequential optimization of the different excited states. Alternative hybrid excited state algorithms include the quantum subsbace expansion (QSE) method,^21,77,78^ in which the Hamiltonian is diagonalized in some subset of the Hilbert space to obtain estimates of low-lying eigenstates; the multistate-contracted VQE method,^79^ which optimizes an entanglement matrix between some approximate excited states obtained from a conventional quantum chemistry calculation; or the witness-assisted variational eigenspectra solver (WAVES),^80^ which uses the von Neumann entropy of states to ascertain whether they are eigenstates of the Hamiltonian.

Another approach to directly target excited states is the folded-spectrum method,^20,36,81^ in which the system Hamiltonian *Ĥ* is replaced by
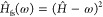
29which has the same eigenstates as the original *Ĥ*. However, the eigenvalues become *E*_fs;*i*_ = (*E*_*i*_ – ω)^2^ so the ground state of *Ĥ*_fs_(ω) becomes the eigenstate with true energy *E*_*i*_ closest to ω. Therefore, by changing ω one can target any stationary state of the system with conventional variational or projective techniques. This method has been previously used in conjunction with FCIQMC^82^ and similar approaches have been used in the conventional Variational Monte Carlo (VMC) community under the name of state-specific variance optimization.^83−87^

In variational folded-spectrum approaches, one minimizes the quantity
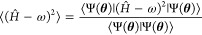
30while in projective approaches one updates the parameters using the residuals
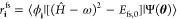
31The algorithms employed are equivalent to the standard ground-state approaches, but the folded-spectrum Hamiltonian is more complex than the original Hamiltonian. Consider a standard chemistry Hamiltonian
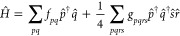
32where *p̂* and *p̂*^†^ are Fermionic creation and annihilation operators, respectively, for spin-orbital *p* and *f*_*pq*_ and *g*_*pqrs*_ are the one- and two-body contributions to the Hamiltonian. The corresponding folded-spectrum Hamiltonian will include up to four-body terms,
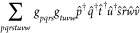
33which will be significantly more expensive to compute than the one- and two-body terms of the original Hamiltonian. The total number of terms in the operator will therefore scale as *N*^8^ in the number of one-electron basis functions, rather than the *N*^4^ scaling of the original Hamiltonian. Due to this significant cost increase, the folded-spectrum technique is currently limited to only small systems.

## 3 Quantum Algorithms

### 3.1 Unlinked Residual Measurement Implementation

We begin by discussing the implementation of quantum circuits to compute the unlinked residual
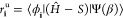
34and the overlap
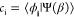
35required by the QMC methods discussed in section 2.3.

As detailed in section 2.3, exponential Ansatz-based QMC algorithms require complex selection schemes to estimate *r*_**i**_^u^, as computing it directly would require storing the full CI wave function, which is not memory-efficient. For large cluster sizes, this often becomes the limiting factor on the speed and rate of convergence of a calculation. Therefore, using a quantum device to compute these terms without the memory overhead would be a significant boon to these methods. This has been shown in the context of auxiliary-field QMC (AFQMC),^88^ for which overlaps with complex trial wave functions can be efficiently calculated using a quantum computer.^89^

**Figure 1:**
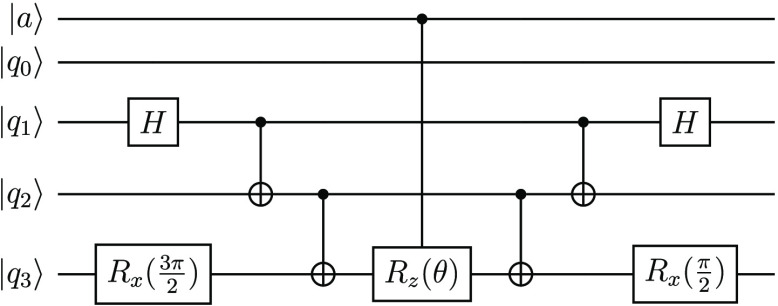
Pauli gadget encoding a controlled version of .

The standard implementation of the UCC Ansatz in the quantum circuit representation is to map Fermionic creation and annihilation operators onto strings of Pauli *X*, *Y*, and *Z* matrices (or gates). There are multiple standard mapping schemes,^90−93^ of which we employ here the Jordan–Wigner approach,^90^ in which
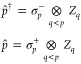
36where *X*_*p*_, *Y*_*p*_, and *Z*_*p*_ denote the usual Pauli matrices acting on the *p*-th qubit, the product of *Z* matrices encodes the required parity to preserve Fermionic anticommutation relations, and σ_*p*_^±^ = *X*_*p*_ ± *iY*_*p*_. Using this mapping leads to the following forms for single and double excitation operators:
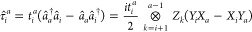
37
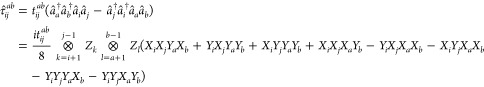
38The standard approach to computing the exponential of each of these strings of Pauli matrices is to use a set of gates known as a Pauli gadget,^94^ a controlled version of which is shown in Figure 1. While this is the approach taken in this work, we note that more compact alternatives have also been devised.^95,96^

**Figure 2:**
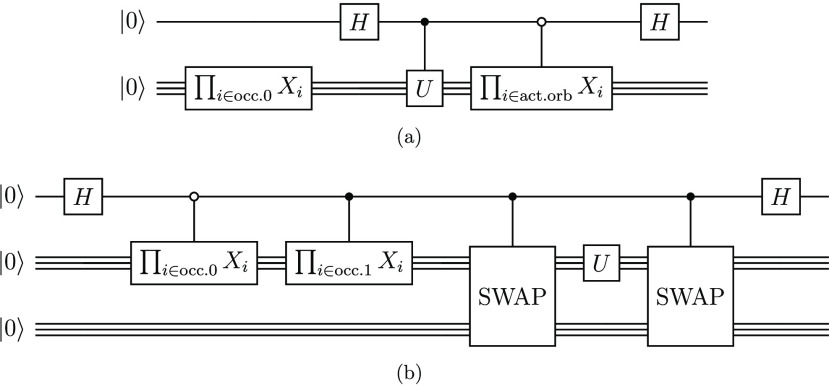
Quantum circuits for measuring the overlap *c*_**i**_ = ⟨ϕ_**i**_|*Ûϕ*_0_⟩. (a) A modified Hadamard test,^97^ which uses a single ancilla qubit and a controlled version of the unitary *Û*. (b) An alternative proposed by Huggins et al.,^98^ which needs (*n*_qubits_ + 1) ancilla but no controlled *Û*. The products of *X* gates go over the occupied orbitals in Φ_*p*_ (denoted occ.*p*) or over the active orbitals in the excitation Φ_0_ → Φ_i_ (denoted act.orb).

As there is no immediate expectation-value form similar to eq 11 for *r*_**i**_^u^ and *c*_**i**_ computing such quantities requires auxiliary qubits. Two alternative circuit constructions to obtain *r*_**i**_^u^ and *c*_**i**_ are given in Figure 2.

The first uses fewer ancilla qubits, but it also requires a controlled version of the unitary *Û*, which decomposes into a larger number of two-qubit gates than its uncontrolled couterpart, regardless of the nature of *Û*. As a significant fraction of NISQ device error is due to two-qubit gates, the second circuit may be preferable on hardware. However, it requires an ancilla register of the same size as the system register, which causes a 2^*n*_qubits_^-fold increase of the size of the qubit Hilbert space, leading to worse performance on quantum simulators. Work presented here therefore employs the circuit in Figure 2 (a).

In the particular case of the Pauli-gadget-based unitary coupled cluster Ansatz, controlled-*Û* only requires one additional controlled rotation gate per Pauli gate string, making it relatively easy to implement. Compared to a given UCC circuit employed in conventional VQE, this approach requires one additional qubit and 2*n*_excit_ + 1 additional gate layers, in order to encode the determinant corresponding to each residual. The method also requires a constant number of additional two-qubit gates for each parameter in the Ansatz. The asymptotic scaling of circuit depth and two-qubit gate count is therefore equivalent to a conventional VQE algorithm employing the same UCC Ansatz.

Labeling the overall state in the qubit register of the circuit in Figure 2 (a) as |Φ⟩, the expectation value of *Z* on the ancilla is given by
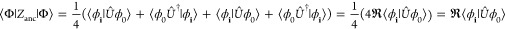
39so ⟨Φ|*Z*_anc_|Φ⟩ = (*c*_**i**_). The *Y*-expectation value similarly gives the imaginary part of the overlap. Additionally, applying the Hamiltonian to the system register and measuring the expectation value gives the corresponding parts of the residual,
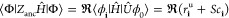
40

In order to do this, the second-quantized Hamiltonian in eq 32 must be mapped onto a corresponding qubit Hamiltonian, which can be done using the same techniques discussed for excitation operators. This leads to an operator of the form
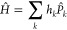
41where *P̂*_*k*_ are strings of Pauli operators. The expectation value in eq 40 can then be rewritten as
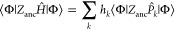
42and each term measured individually. The cost of measuring each residual therefore scales with the number of terms in the Hamiltonian, like the total energy calculation. Residuals computed in this way can be employed in a projector method based on eq 21, leading to an unlinked PQE algorithm. Given noiseless, exact values for *r*_**i**_^u^ and setting the shift to track the instantaneous projected energy, such an algorithm would converge directly to the ground state, without need for further Monte Carlo estimation. However, expectation values obtained from a realistic quantum device are subject to at least finite-sampling noise. Therefore, it is perhaps wiser to consider the measured values of *r*_**i**_^u^ as stochastic estimates of the underlying quantities and therefore use them in a QMC-like algorithm. The resulting MC-PQE method is outlined below:

1. On a classical machine, represent the wave function *Û*(**θ**)|ϕ_0_⟩ as a distribution of walkers with populations *N*_0_ corresponding to |ϕ_0_⟩ and *N*_*i*_ = θ_*i*_*N*_0_ corresponding to each parameter.
2. Encode the wave function *Û*(**θ**)|ϕ_0_⟩ on a quantum device and measure the residuals *r*_**i**_^u^. This is done by obtaining the parameters **θ** from the classical distribution as  and substituting them into the circuit in Figure 2 (a). Measuring each residual is equivalent to an energy measurement. As in VQE, this requires individual measurement of each (noncommuting) term in eq 41, scaling as *n*_qubit_^4^. The number of residuals is equal to the number of parameters in the employed Ansatz.
3. Update each *N*_**i**_ = *N*_**i**_ – *δβN*_0_*r*_**i**_^u^.
4. Store the projected energy *E*_proj_.
5. Once a threshold population *N*_tot_ = ∑_**i**=0_^*N*_param_^ *N*_**i**_ has been reached, allow the shift to vary to maintain this population constant. In the original FCIQMC algorithm this is done by^32^
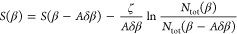
43where *N*_tot_ is the total walker population, *A* is the number of steps the shift is updated after, and the damping parameter ζ controls the rate at which the shift responds to changes in population. More recent update procedures have been developed that lead to finer population control and more rapid convergence.^99^
6. Repeat steps 2–5 until sufficient samples of *E*_proj_ and *S* have been accumulated to obtain accurate Monte Carlo estimates.

On the one hand, as discussed in section 4, this approach has a series of benefits relative to a “deterministic” PQE approach, as it is resilient to noise in the quantum measurement, allowing the use of low shot numbers to obtain accurate results. Additionally, the shift provides a second independent estimator for the energy which can be used to more clearly identify when convergence has been reached. On the other hand, it is more efficient than a fully classical Quantum Monte Carlo algorithm as it both removes the need for cluster sampling and merges the death and spawning steps into a single process. Additionally, this joint process can be seen as originating from the whole wave function, which removes the dependence of the algorithm on the total walker population.

One of the main benefits of QMC methods over conventional algorithms is that they take advantage of sparsity in the Hamiltonian to store a compressed version of the wave function, thereby decreasing classical memory overheads. In the following section we explore these QMC-inspired ideas to further decrease the memory or quantum resource costs of the algorithm detailed above.

### 3.2 Stochastic Approximations

#### 3.2.1 Sampling the Hamiltonian

When computing the residuals *r*_**i**_^u^ one must in principle measure each of the terms of the qubit Hamiltonian separately, as they require different postrotations to be applied to the circuit before measurement. However, they can be further grouped^37,100^ as
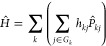
44where [*P̂*_*ki*_, *P̂*_*lj*_] ∝ δ_*kl*_. Terms in the same group *G*_*k*_ commute, so they may be measured simultaneously using the same postrotations. Nevertheless, in the general case, this does not reduce the operator to a single group, so a significant number of terms still need to be measured independently. Additionally, optimal grouping of Pauli terms is in general a hard problem.^101^ For the two-body quantum chemistry Hamiltonian there is a simple heuristic partitioning, based on the qubitwise commutativity of Pauli strings. This leads to one large group comprising the identity string and all strings containing only *Z* gates and many small groups of strings containing some *X* and *Y* gates. Significant work has gone into further optimizing the number of measurements required to obtain Hamiltonian expectation values, including modifying the partitioning between groups^30,31,102^ or the Pauli strings themselves^29^ to lower variance, or using (randomized) measurement techniques based on the classical shadows approach.^28,103−105^

Regardless of the means by which groups are obtained, one can employ an approach similar to that used in classical QMC algorithms and only select a subset of the groups to measure at each time-step. This decreases the number of circuit measurements needed per time-step, but introduces further noise in the estimate of *r*_**i**_^u^. However, provided no systematic bias is caused, this noise will be averaged out over the course of the imaginary-time propagation.

There exist various possible Hamiltonian term selection schemes, the simplest of which would be to sample uniformly from the set of Pauli terms or groups. Many of these terms will have negligible weights, however, so in order to better capture the action of the Hamiltonian in a small number of samples, some form of importance sampling is recommended. One straightforward approach is to select each Pauli group with probability proportional to the group weight *W*_*k*_ = ∑_*j* ∈ *G*_*k*__|*h*_*kj*_|,
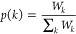
45

For most quantum chemistry Hamiltonians investigated in this paper, we find that, when using qubitwise commutativity to partition the terms, the weight of the group containing the empty Pauli string and all *Z*-only strings, which we will label *G*_0_, strongly dominates the resulting probability distribution. Therefore, under this selection scheme, it is likely that for small numbers of samples it will be *over-* rather than *under*sampled. We also note that this term is qualitatively different from the other groups, as it describes the diagonal action of the Hamiltonian, while the others all correspond to off-diagonal contributions. Therefore, to obtain a more balanced description, we propose a scheme in which the diagonal group *G*_0_ is always evaluated, while the other groups are selected with probability
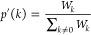
46

#### 3.2.2 Sampling the Wave Function

UCC is the natural candidate Ansatz for this type of quantum-enhanced QMC algorithm. One of the main challenges with using this Ansatz on NISQ devices is that it leads to deep circuits, with execution times that easily exceed the coherence times for current machines. We have previously shown that by using insight from a fully classical UCCMC calculation,^34^ one can significantly shorten circuits while preserving accuracy by removing low-weight excitors from the Ansatz.^106^

This circuit simplification can be done on-the-fly during the propagation, by stochastically rounding the parameter values before encoding the wave function,
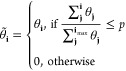
47where *p* is a uniform random sample from the interval [0, 1) and the parameters θ_**i**_ are ordered by decreasing amplitude. Once again, this is expected to increase fluctuations in the estimators at each time-step. The issue of potential systematic biases is explored further in section 4.

#### 3.2.3 Spawning

In the methodology detailed above, all parameters are updated at every time-step. However, one can take the approach commonly employed in QMC algorithms and only update a stochastically selected subset of the populations. In particular, for spawning in conventional QMC algorithms one can use a variety of excitation generation algorithms.^107−110^

In principle, one could spawn onto connected determinants uniformly. However, the spawning probability is proportional to  (see eq 22), so for appropriate importance sampling of this process it is desirable that *p*_gen_(**i|j**) ∝ *H*_**ij**_. Different algorithms have been designed to approximately achieve this, using heat-bath sampling^107,110^ or mathematical bounds on the value of *H*_**ij**_.^108^

With the residuals computed according to the quantum algorithm in section 3.1, death and spawning are no longer separate processes, so the parameters to be updated should be generated with
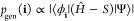
48

If we write out the Fermionic Hamiltonian as *Ĥ* = ∑_*k*_ *h*_*k*_^F^*F̂*_*k*_, where *F̂*_*k*_ is some Fermionic excitation operator and (nonuniquely) label the excitation between determinants ϕ_**i**_ and ϕ_**j**_ as *F̂*_**ij**_, then
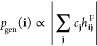
49Sampling the distribution *p*_gen_ is nontrivial even with access to a quantum computer and a qubit representation of the wave function and Hamiltonian. In particular, the circuits described in Figure 2, which can be used to estimate the overlap itself, cannot be used for this purpose.

**Figure 3:**
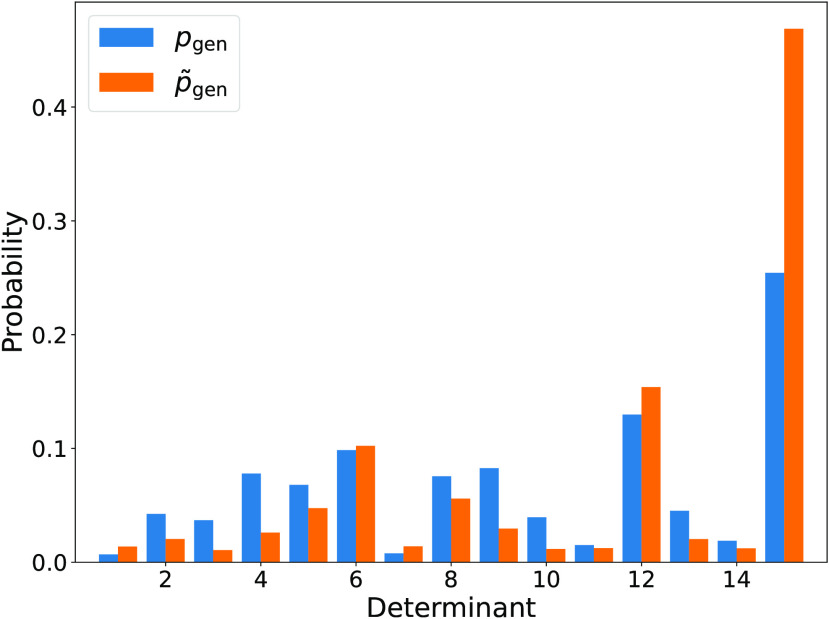
Excitation generation probability according to eq 49 and eq 50 for the H_4_ molecule, using an approximate ground state wave function. *p̃*_gen_ is a better approximant for *p*_gen_ than the uniform distribution. As a metric, the Kullback–Leibler divergence^111^ of *p̃*_gen_ with respect to *p*_gen_ is 0.118, while that of the uniform distribution is 0.541.

However, one can efficiently generate determinants with probability
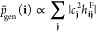
50by preparing the state |Ψ⟩, applying one of the operators *P̂*_*k*_ corresponding to each *F̂*_*k*_ to it with probability  and then measuring the qubit register. While this is not a perfect importance sampling of the Hamiltonian coupling, it will better match its minima and maxima and should therefore represent an improvement over uniform sampling. An example for the H_4_ molecule is shown in Figure 3. These circuits do not require ancilla or additional controlled operations, so they are less costly to implement than those needed to compute the residuals. The number of shots needed is equal to the number of determinants one wants to generate, so they would add little computational overhead.

While stochastic parameter updates may be valuable for larger systems, for the problems considered in this paper it is easy to store and cycle through all the relevant parameters and therefore probabilistic spawning is not employed.

### 3.3 Folded Spectrum Considerations

All the methods described above can equally be applied to the folded spectrum Hamiltonian, by replacing the residual with that in eq 31. As the squared Hamiltonian operator contains many-body terms of higher order than the original Hamiltonian, this will lead to more terms in the Pauli string representation of the corresponding qubit operator as well. However, some of these will commute with the terms in the original operator, so the number of different groups will see a smaller increase. Nevertheless, for nontrivial systems, this leads to a potentially insurmountable increase in required computation, even with efficient Hamiltonian term grouping methods. The possibility of sampling the Hamiltonian is therefore particularly promising in this case as a means to mitigate the additional costs.

As molecules are pulled apart electronic states approach one another and often become degenerate in the fully dissociated limit. Therefore, if one is searching for two different states which are close in energy (which will remain so after the folding procedure), noise in the wave function representation becomes potentially problematic as it may be sufficient to induce a switch from one state to another. In the case of ground state calculations, it has been observed in conventional CCMC that, particularly in more strongly correlated regimes, large fluctuations will sometimes cause a calculation to collapse onto a different, often unphysical, state.^112,113^ This is made possible by the nonlinear structure of the coupled cluster equations, which allow for multiple solutions, not all of which have a configuration interaction correspondent.^114,115^ Therefore, the range of stochastic techniques that can be used in this scenario may be more limited than in ground state calculations.

## 4 Results

All results in this paper are obtained from state-vector or shot-based quantum circuit simulation using the t|ket⟩ platform.^116^ Necessary atomic orbital Hamiltonian integrals were obtained from PySCF^117^ and FCI calculations were performed using HANDE-QMC.^118^ All calculations used the UCCSD Ansatz. The main systems considered are linear hydrogen molecules up to H_8_ in the STO-3G basis set,^119^ which serve as a useful proof of concept for the methods described here. These molecules are small enough that the corresponding Fock space vectors can be manipulated on a classical computer, while covering a range of chemical behavior. Shot-based simulations are obtained by taking *N*_shots_ samples from the probability distribution corresponding to the wave function encoded in a given quantum circuit.

In order to reduce computation time required for larger systems, shot-based simulation is at times replaced by state-vector simulation with added Gaussian noise of mean μ = 0 and standard deviation σ. The average behavior of such simulations should be very similar to true shot-based results for large enough *N*_shots_, but it will fail to capture some of the catastrophic failures of the very low shot count regime.

Average values of the shift *S* and *E*_proj_ are obtained by a reblocking analysis^120^ of data obtained in the steady-state regime of calculations, using pyblock.^121^

### 4.1 Ground State Calculations

We begin our investigation into the effects of sampling noise on the MC-PQE algorithm by carrying out calculations using the circuit in Figure 2 (a), sampled with 10^1^ to 10^4^ shots for each Pauli term in the Hamiltonian.

**Figure 4:**
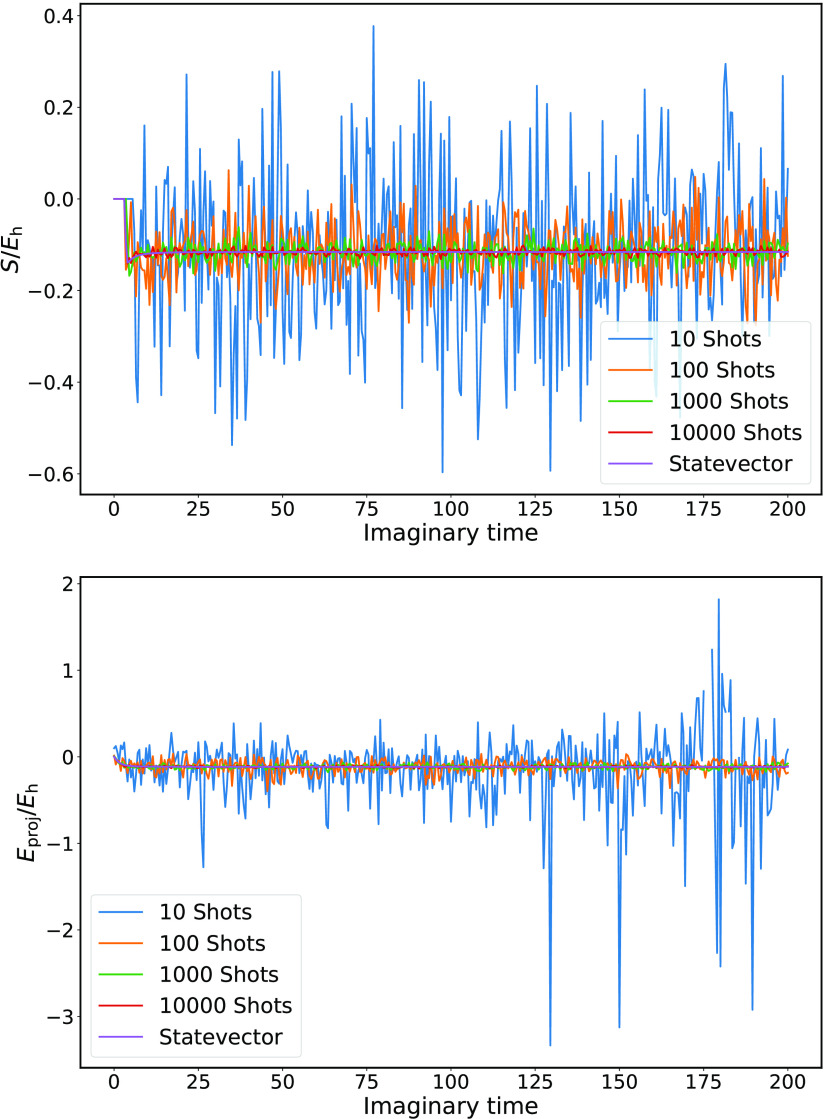
Shift (top) and projected correlation energy (bottom) as a function of imaginary time, obtained from 10^1^–10^4^ samples of the residual circuits for each Pauli term in the Hamiltonian, for H_3_^+^ at *r*_HH_ = 2.0 Å. Noise decreases as the number of shots increases.

Representative examples of MC-PQE energies over the course of calculations with differing numbers of shots for H_3_^+^ at a bond length of *r*_HH_ = 2.0 Å are shown in Figure 4. The instantaneous energy estimators display significant fluctuations, although, unsurprisingly, the noise decreases as the number of shots is increased.

There is a qualitative change in the behavior of the projected energy using *N*_shots_ = 10, with some samples deviating significantly from the expected mean. This is effectively due to the underestimation of the denominator in the expression of *E*_proj_ (see eq 24), which leads to divergences in this quantity. There is therefore a lower bound on the number of shots needed to obtain a stable calculation, which is dependent on the overlap ⟨ϕ_0_|Ψ(β)⟩. The final estimators of the energy, obtained by averaging over the instantaneous energies once the calculation has reached its steady-state regime are given in Table 1 and display the expected lower variances as the number of shots increases.

**Table 1:** Mean and Standard Deviation of the Shift and Projected Correlation Energy as the Number of Shots Is Increased, for H_3_^+^ at *r*_HH_ = 2.0 Å^a^

*N*_shots_	⟨*S*⟩	σ(*S*)	⟨*E*_proj_⟩	σ(*E*_proj_)	*E*_FCI_
10	–0.111	0.009	b	b	–0.11586
100	–0.116	0.003	–0.114	0.004	
1000	–0.1167	0.0009	–0.117	0.001	
10000	–0.1163	0.0003	–0.1155	0.0004	

a All energies are given in Hartree.

b As can be seen in Figure 5, the projected energy with only 10 shots diverges at this bond length, so no estimates can be obtained.

Average values for the estimators *S* and *E*_proj_ along the binding curve of H_3_^+^ are given in Figure 5. At large bond-lengths, we observe that divergences in the instantaneous values of the estimators due to undersampling translate into divergences of the overall estimator, with the projected energy more sensitive to this than the shift. This behavior is not unexpected. As the bond length increases, the system becomes increasingly strongly correlated and therefore the weight of the reference determinant ϕ_0_ decreases, requiring more sampling to estimate appropriately.

**Figure 5:**
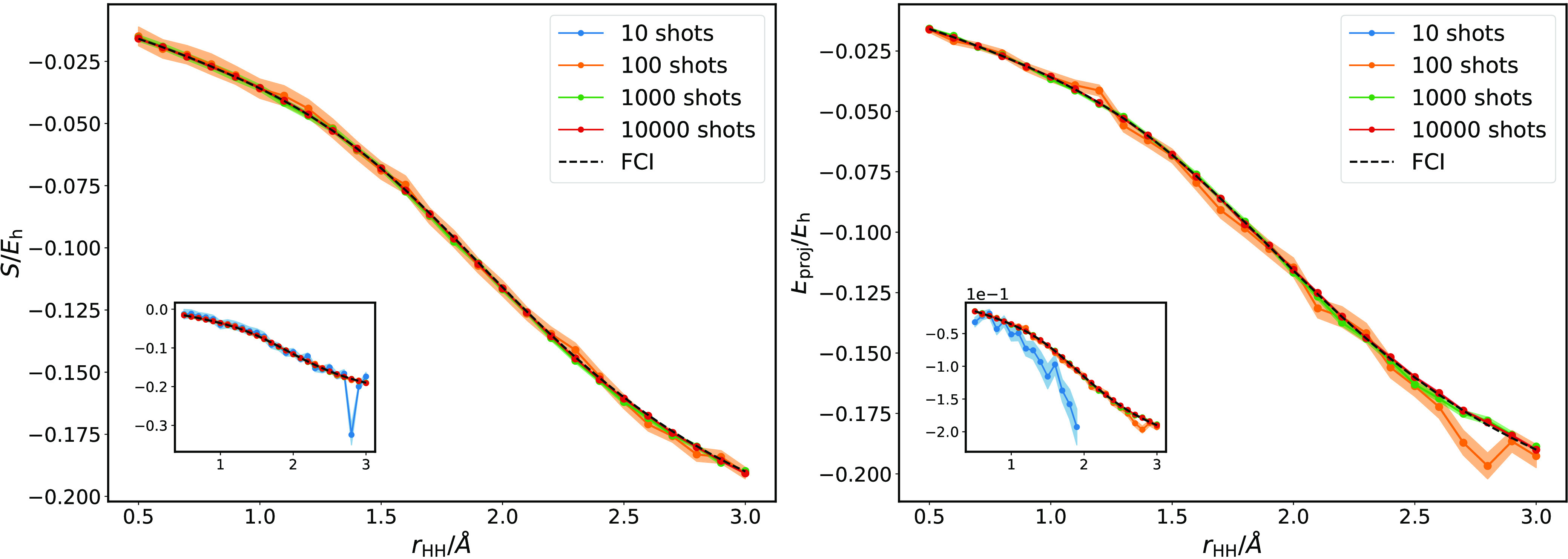
Shift (left) and *E*_proj_ (right) estimates of the correlation energy of linear H_3_^+^ over the range *r*_HH_ = 0.5–3.0 Å. The insets include the estimators obtained with only 10 shots per residual estimation. Deviation from the true correlation energy increases as the number of shots is decreased, with deviations more pronounced in the projected energy and at larger bond lengths. This correlates with an increase in strong correlation and lowering of the overlap of the ground state wave function and the Hartree–Fock reference, ⟨ϕ_0_|Ψ(β)⟩.

It is therefore the case that more strongly correlated systems will require more sampling than weakly correlated ones. This follows well-known trends in the difficulty of solving quantum chemical problems in a classical setting and we can offer a series of ways to alleviate the issue. First, in the absence of noise, the overlap ⟨ϕ_0_|Ψ(β)⟩ decreases monotonically with β. Therefore, it will be possible to use fewer shots at short imaginary times and increase the shot count as the calculation progresses, thereby accelerating convergence to the steady state. For most Monte Carlo calculations, however, the majority of the time is spent sampling this state, which cannot benefit from the early reduction in shots.

Second, different numbers of shots can be allocated to different quantities of interest. In this case, one could run a calculation with relatively small numbers of shots for the residuals, but a large number of shots for the reference overlap estimation. As the system size increases, this will represent a smaller fraction of the computational effort needed, thereby allowing calculations that are effectively as shot-efficient as ones with the lowest number of shots. This approach could be further refined by allocating different shot numbers to different residuals as well, taking into account their expected magnitudes. This would allow for a more efficient distribution of computational effort, such that more significant contributions are estimated more accurately.

Another standard computational chemistry approach would be to replace the Hartree–Fock reference wave function in the projected energy with a more complex, multideterminental quantity |Ψ_T_⟩ which is hoped to have larger overlap with the true ground state, giving a new projected energy of the form
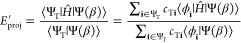
51

On a classical machine, this would require a linear increase in computation, however in the current scheme, all terms in both the numerator and denominator are already computed as part of the propagation. We test this for *H*_3_^+^, which in a minimal basis has two electrons in three orbitals, using a trial wave function of the form
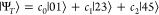
52which includes the dominant configurations at dissociation, where |*ij*⟩ is the Slater determinant with spin-orbitals *i* and *j* occupied. The orbitals are labeled in order of increasing energy, with even numbers corresponding to *m*_*s*_ = 1/2 and odd numbers to *m*_*s*_ = −1/2 orbitals. This is insufficient to ensure convergence of the extremely undersampled 10-shot calculation toward dissociation, but it does reduce the standard error in 100-shot calculations by a factor of approximately 1.5.

Finally we compare the performance of MC-PQE with conventional PQE and with VQE, performed using the Simultaneous Perturbation Stochastic Approximation (SPSA) optimization algorithm,^122^ which has been shown to outperform other optimizers for noisy variational optimization.^123^ In Table 2 we compare the average error in the ground state energy obtained by VQE, PQE, and MC-PQE across the symmetric stretch of the H_3_^+^, H_4_, H_6_, and H_8_ chains, for bond lengths in the range 1.0–3.0 Å. All errors are relative to the result of a noiseless L-BFGS optimization of the UCCSD energy. For the two smaller systems, calculations are performed using explicit sampling, while for the larger systems Gaussian noise is used to simulate finite sampling noise. The VQE calculations are run for a fixed number of iterations. For H_3_^+^, MC-PQE is run for 250 iterations. For H_4_, it is run for up to 1000 iterations, with reblocking analyses run every 50 iterations. The reported energy errors and iteration numbers are computed using either the first instance of the variational energy estimator error converging under 1mEh or the best estimate available over the course of the propagation. The same procedure is employed for H_6_ and H_8_, with MC-PQE run for up to 2000 iterations. The number of PQE iterations and samples is chosen to make errors comparable to MC-PQE. VQE and PQE calculations are repeated 5 times to obtain statistical bounds on the errors. For MC-PQE such bounds can be obtained in the course of a single calculation.

**Table 2:** Comparison of VQE, PQE, and MC-PQE Results for Hydrogen Chains^a^

	H_3_^+^					
Method	*n*_q_	*n*_2q_	*n*_shots_	*n*_iter_	*n*_total_	Δ*E*
VQE	6	128	10000	1000	5.00e8	3(2)^b^
PQE	6	128	10000	50	1.13e8	1(2)
MC-PQE	7	152	100	250	3.13e6	1(1)

	H_4_					
Method	*n*_q_	*n*_2q_	*n*_shots_	*n*_iter_	*n*_total_	Δ*E*
VQE	8	736	10000	1000	2.02e9	5(2)^b^
PQE	8	736	10000	111	3.25e9	1(2)
MC-PQE	9	832	500	480	3.64e8	1(1)

	H_6_					
Method	*n*_q_	*n*_2q_	σ	*n*_iter_	*n*_total_	Δ*E*
VQE	12	4560	0.0001	2000	2.40e10 *n*	200(80)^c^
PQE	12	4560	0.0001	49	3.50e10 *n*	2.8(0.2)
MC-PQE	13	4996	0.01	1080	3.89e7 *n*	3(3)

	H_8_					
Method	*n*_q_	*n*_2q_	σ	*n*_iter_	*n*_total_	Δ*E*
VQE	16	18560	0.0001	1000	4.21e10 *n*	700(200)
PQE	16	18560	0.001	46	3.57e9 *n*	6(2)
MC-PQE	17	19285	0.01	1010	3.93e8 *n*	6(5)

a *n*_q_ is the number of qubits and *n*_2q_ the number of two-qubit gates in each circuit. Each method was run for an average of *n*_iter_ iterations, using *n*_shots_ measurements per Pauli string measured at each step or Gaussian noise with standard deviation σ. VQE and PQE calculations were repeated 5 times to obtain statistical error bounds. *n*_total_ is the total number of measurements required. For VQE with SPSA optimization, this is 2*n*_iter_*n*_Hamil_*n*_shots_, for PQE it is (2*n*_param_ + 1)*n*_iter_*n*_Hamil_*n*_shots_, while for MC-PQE it is (*n*_param_ + 1)*n*_iter_*n*_Hamil_*n*_shots_. Δ*E* is the average absolute error over the binding curve, in milliHartree. For a fair comparison, a variational estimate of the energy is also computed for MC-PQE.

b Outliers with energy errors above 100 mE_h_ were removed.

c Outliers with energy errors above 1000 mE_h_ were removed.

First, we note the results of the calculations presented in this table could undoubtedly be further improved by allocating additional computational resources. Nevertheless, we find that in all cases explored here, for a given error, MC-PQE requires at least 1 order of magnitude fewer samples to obtain comparable results to conventional PQE. This is also true in comparison to VQE, where the latter converges well. For the larger H_6_ and H_8_ systems, VQE encounters convergence problems, which we do not observe for either version of PQE. This is to be expected due to the imaginary-time projection underlying these methods. As described above, MC-PQE requires one ancilla qubit and an increased number of two-qubit gates, but the relative additional cost decreases with system size, as can also be seen from Table 2.

We move on to consider stochastic implementations of the projection algorithm itself.

**Figure 6:**
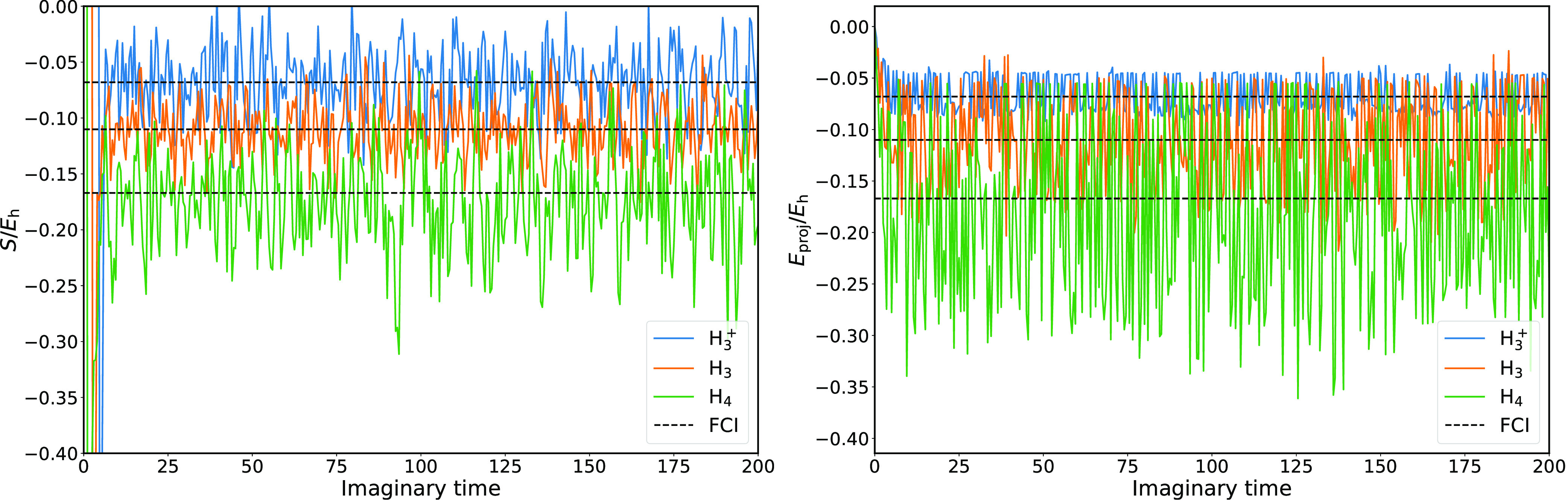
Shift (left) and projected correlation energy (right) as a function of imaginary time, obtained from a stochastically represented wave function for H_3_^+^ (blue), H_3_ (orange), and H_4_ (green) at *r*_HH_ = 1.5 Å. The noise in the shift is roughly system-independent, whereas the variance of *E*_proj_ increases with its absolute value.

We begin by considering the effects of stochastic sampling of the wave function and Hamiltonian independently, while doing the projection itself deterministically. Additional example MC-PQE trajectories are given in the Supporting Information. Figure 6 shows trajectories computed using deterministic projection on a stochastically rounded wave function for H_3_^+^, H_3_, and H_4_, with corresponding statistical properties given in Table 3. H_2_ is not considered here as it only has one parameter in the wave function, so there is no rounding to be done. While the noise introduced into the shift by this procedure is roughly constant and system-independent, in itself a desirable property, the noise in *E*_proj_ scales linearly with the absolute magnitude of the correlation energy, as can be seen from the right panel of Figure 6. This is unsurprising since if all amplitudes are rounded down, we expect the correlation energy to be zero. Nevertheless, for all three systems we obtain stable estimators for the correlation energy. For the H_3_ and H_3_^+^ systems, for which the UCCSD Ansatz is exact, the results are within error bars of the FCI value. For H_4_, there is an error of 10 milliHartree in the correlation energy estimators. This can be significantly reduced by computing the variational rather than projected energy estimator.^34^

**Table 3:** Mean and Standard Deviation of the Shift and Projected Correlation Energy Obtained from the Deterministic Statevector Propagation of Stochastically Rounded Wavefunctions for Systems of Increasing Size^a^

System	⟨*S*⟩	σ(*S*)	⟨*E*_proj_⟩	σ(*E*_proj_)	*E*_FCI_
H_3_^+^	–0.0688	0.0005	–0.068	0.001	–0.06803
H_3_	–0.113	0.001	–0.1107	0.0009	–0.11011
H_4_	–0.180	0.003	–0.176	0.002	–0.16701

a All systems have bond lengths of *r* = 1.5 Å, and all energies are given in Hartree.

We then consider propagating the full wave function with a stochastically sampled Hamiltonian in which only *N*_Hamil_ commuting Pauli groups are included at each time-step. Corresponding values of the estimators for such calculations are given in Table 4. Once again, as expected, variance decreases with the number of terms included and we note that propagation under the action of the stochastically sampled Hamiltonian does not introduce any additional bias to the estimators. Noise in the shift fully depends on the details of the stochastic trajectory, but in order to minimize the variance of *E*_proj_ we use the full Hamiltonian at each step. As this is equivalent to a single residual computation, the relative cost of employing the full Hamiltonian at this stage decays with system size. However, the projected energy could also be estimated using the sampled Hamiltonian at each step, decreasing computational overhead at the cost of increased sampling noise.

**Table 4:** Mean and Standard Deviation of the Shift and Projected Energy Obtained from Linearized Imaginary Time-Propagation of the H_3_^+^ Wavefunction at *r* = 2.0 Å, Using Stochastically Sampled Hamiltonians with Increasing Numbers of Terms Selected per Step^a^

*N*_Hamil_	⟨*S*⟩	σ(*S*)	⟨*E*_proj_⟩	σ(*E*_proj_)	*E*_FCI_
2	–0.115	0.003	–0.125	0.007	–0.11586
3	–0.116	0.001	–0.121	0.004	
4	–0.117	0.001	–0.121	0.004	
5	–0.1137	0.0007	–0.115	0.004	
6	–0.1133	0.0007	–0.112	0.003	

a All energies are given in Hartree.

Given the data presented above, we are satisfied that shot-based simulation, stochastic wave function rounding and Hamiltonian sampling are all compatible with MC-PQE and do not introduce significant biases into the results of a simulation. They do however all increase the noise present, requiring, much like classical QMC methods, a relatively long period of steady-state propagation to converge the desired quantities to the intended accuracy.

There are further ways to reduce the noise in the simulation which do not require increased sampling. One such method is *shift damping*,^32^ which entails decreasing the parameter ζ in eq 43. This makes the shift less responsive to changes in population, thereby directly decreasing its variance. As the value of the shift affects the continued propagation of the wave function, we expect that, once the steady-state has been reached, less variability of the shift will translate to less variability of the wave function and therefore one might expect less noise in the projected energy as well.

As an example, we compare the shift and projected energy in calculations with different numbers of shots and different degrees of shift damping for *H*_3_^+^. While a 10-fold decrease in ζ is very effective in reducing shift noise (σ_damped_/σ = 18% (1000 shots), 20% (100 shots)), we see no effect in the projected energy (σ_damped_/σ = 101% (1000 shots), 102% (100 shots)). See the Supporting Information for example MC-PQE trajectories.

**Figure 7:**
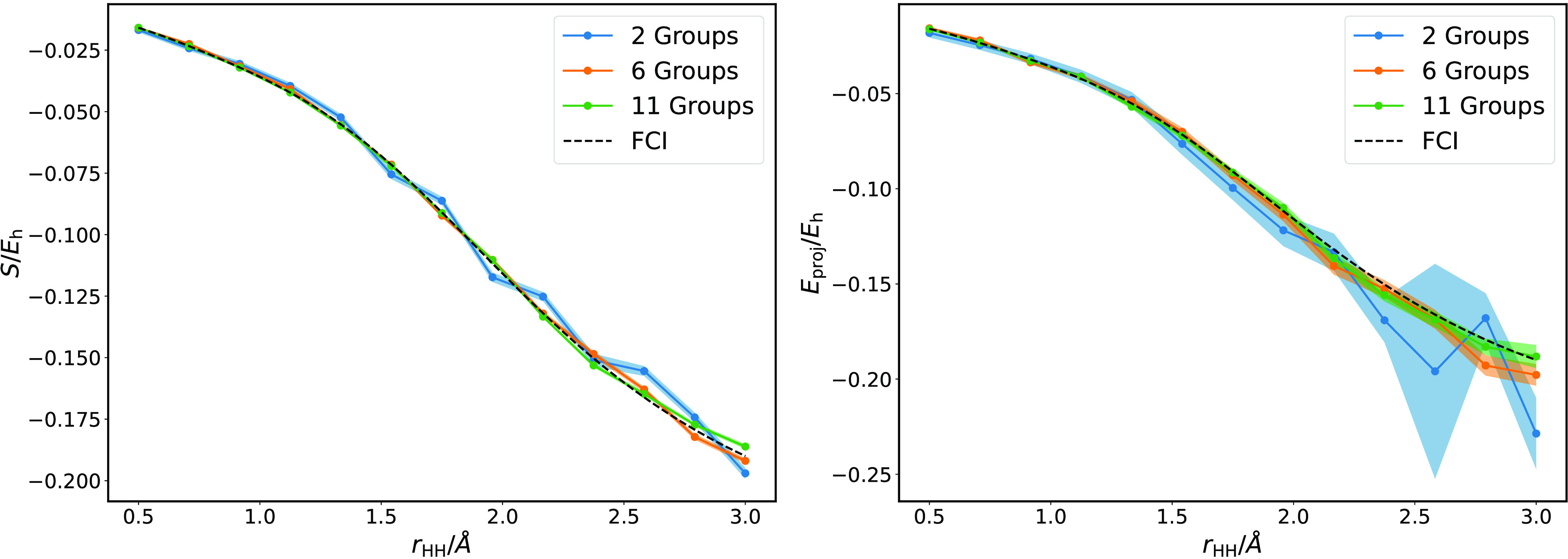
Shift (left) and projected correlation energy (right) estimators for the H_3_^+^ correlation energy, obtained using a sampled Hamiltonian with only 2–11 Pauli groups selected at each time-step, a stochastically rounded wave function, and 1000 shots per residual.

Finally, we combine shot-based simulation with stochastic wave function rounding and Hamiltonian sampling. *H*_3_^+^ binding curves obtained with the fully stochastic algorithm are given in Figure 7. Simulations remain well behaved even with multiple sources of noise. We also note that the systematic increase in error due to stochastic rounding of the wave function appears to be partially canceled out by other noise. As noted in the case of pure finite sampling noise, more highly correlated regimes require better sampling to achieve the same level of accuracy.

We also apply this stochastic PQE algorithm to first row mono- and dihydrides over a range of geometries, with results summarized in Table 5. The cases studied include the dissociation of LiH and HF, the symmetric stretch of H_2_O at its equilibrium bond-angle and the *C*_2*v*_ insertion of Be into the H_2_ bond to form BeH_2_.^124^ Geometries for HF and H_2_O match those from ref (125), BeH_2_ geometries follow the trajectory described in ref (124) and the LiH bond lengths considered are *r*_LiH_/Å= {0.5, 1.0, 1.5, 2.0, 2.5}. The STO-3G basis set was used for all molecules. When using the same calculation parameters for all systems, it is unsurprising that errors are larger for more complex molecules. However, we note that by stochastically rounding the wave function and only sampling 20 Hamiltonian groups for each system, similar errors to the pure finite sampling case are observed. In all cases, the results correspond to relatively short propagation times and errors could be reduced by running the calculations further.

**Table 5:** Maximum Absolute Error (MAE) and Non-Parallelity Error (NPE) in milliHartree for Monte Carlo PQE in Some Frozen-Core Second Row Hydrides, Compared to Fully Deterministic PQE^a^

	MAE		NPE		MAE (10)		NPE (10)		MAE (20)		NPE (20)	
System	S	E	S	E	S	E	S	E	S	E	S	E
LiH	0.5	0.3	0.6	0.6	2	1	4	1.6	2	0.7	2.7	1
BeH_2_	0.6	0.9	0.5	0.9	2	3	5	6	2	3	4	4
H_2_O	6	4	6	6	42	76	53	72	9	5	10	5
HF	9	4	14	4	10	13	18	20	6	10	6	14

a These systems have 125 (LiH and HF) and 313 (H_2_O and BeH_2_) Hamiltonian groups, respectively. Results in all columns have Gaussian noise with σ = 0.001 added to simulate sampling noise, and are obtained from runs of 2000 imaginary time-steps *δβ* = 0.2. The first set of results (columns 1–4) has no additional stochasticity. The second (5–8) and third (9–12) sets use stochastic wavefunctions and 10 or 20 sampled Hamiltonian terms per step, respectively. The error converges toward the pure sampling error with few sampled Hamiltonian groups. For all methods, errors could be further decreased by using longer propagation times.

### 4.2 Excited States

**Figure 8:**
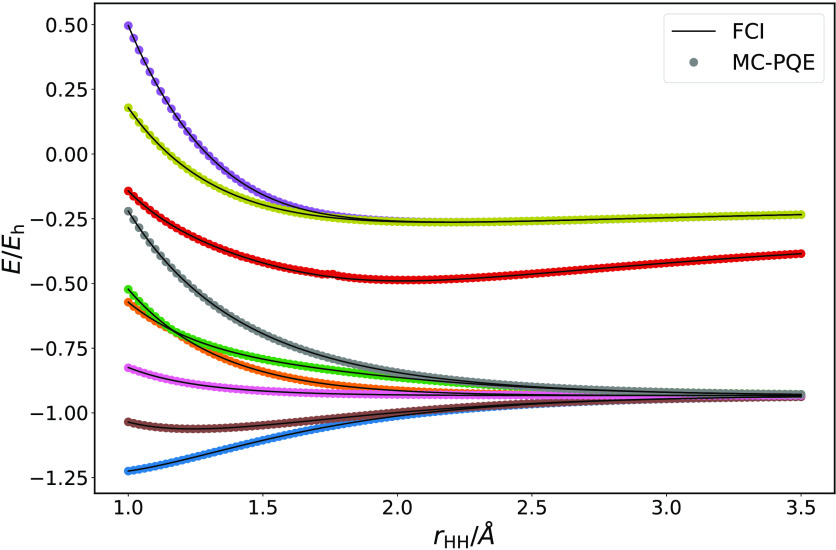
Statevector-simulated folded-spectrum energies for the H_3_^+^ molecule in the STO-3G basis set, using a projective eigenvalue solver. The Slater determinants used as initial references for each excited states are, in increasing energy order of the resulting state: |01⟩, |03⟩, |12⟩, |23⟩, |05⟩, |14⟩, |25⟩, |34⟩, |45⟩, following the same conventions defined for eq 52.

As described in Sec II D, the algorithms above can also be applied to excited states by employing the modified Hamiltonian in eq 29. The results of example folded-spectrum calculations performed using exact statevector simulations for H_3_^+^ are given in Figure 8. In order to easily converge to the desired state, the calculations are initialized at the first geometry (shortest *r*_HH_) with different reference determinants and values of ω close to their energies. States are then tracked along the binding curve by using the parameters and energies of the previous point to initialize the wave function and ω value for the current point. This allows smooth tracking of the states over the whole range of geometries considered.

**Figure 9:**
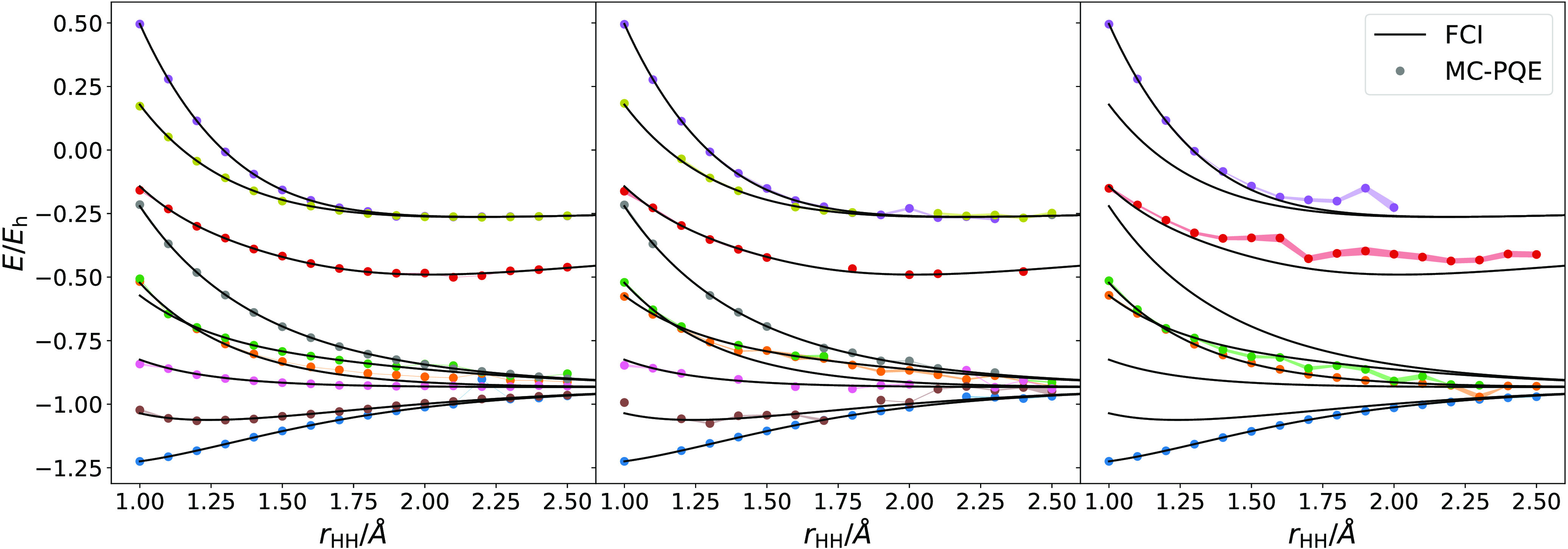
Folded-spectrum energies for the H_3_^+^ molecule in the STO-3G basis set, using a projective eigensolver and (left) Gaussian noise with σ = 0.01, (middle) Gaussian noise with σ = 0.01 and 10 Hamiltonian groups sampled at each step, and (right) Gaussian noise with σ = 0.01 and a stochastically rounded wave function.

We investigate introducing noise into these simulations, first in the form of pure sampling noise due to shot-based simulation. Results obtained using Gaussian noise with σ = 0.01 for H_3_^+^ are shown in the left panel of Figure 9. We note that, even with relatively realistic finite sampling noise levels, convergence is still very good, with obtained states closely following their FCI counterparts.

However, not all instances of noise are as benign in this scenario, as can be seen in the right panel of Figure 9, which shows results for the totally symmetric states of H_3_^+^ obtained with stochastically rounded wave functions. In this case, particularly for the highly excited states, the rounding of the wave function leads to unphysical behavior. The third excited state is most strongly affected, at points tending toward converging onto the highest excited state instead. One must note that because previous values of the energies are used as ω values at subsequent points, once a sufficiently large deviation occurs it is likely to remain or be amplified. This could be avoided by running independent calculations at each geometry, but a new problem of estimating *a priori* a good initial wave function and ω is introduced.

The main limitation to using the folded spectrum method is the increase in terms in the squared Hamiltonian compared to the original operator. However, the Monte Carlo stochastic sampling of the Hamiltonian described in section 3.2 provides an effective means of sampling the folded spectrum operator. We find (see middle panel of Figure 9) that the same number of sampled Hamiltonian terms can be used per step as in standard ground state calculations to generate generally good quality excited state energies, although once again, where states are close in energy this may lead to convergence onto different states than the one targeted. This behavior has been observed for conventional PQE as well, in the presence of noise.^64^ This significantly expands on the range of systems for which folded spectrum methods are tractable.

## 5 Conclusion

In this paper, we present a Quantum Monte Carlo-informed approach to projective quantum eigensolver algorithms. We begin by noting that rather than attempting to obtain the true ground state of a system to within the desired accuracy directly, once propagation has converged, we can obtain highly accurate estimates by classically averaging over samples obtained at different times over the course of imaginary time propagation. This allows us to use low shot counts without compromising final accuracy, leading to a significant reduction in the number of samples relative to comparable conventional VQE and PQE calculations. Notably, this reduction is due to an effective increase in the target variance of the measured observable, and therefore orthogonal to potential improvements due to more effective utilization of the measurement information itself. Therefore, this method should be usable in conjunction with efficient measurement techniques such as those based on classical shadows to great effect.

We go further to decrease the quantum computational overhead, through stochastic rounding of the wave function and probabilistic sampling of the Hamiltonian. These procedures lead to shallower circuits and fewer shots required per step, but also generate noisier projectors. However, we observe that the Monte Carlo energy estimators employed are generally resilient to this additional noise, generating comparable accuracy to the deterministic projector, provided the resulting truncation at each step is not too drastic. In practice, for first row hydrides we observe that as few as 10% of the Hamiltonian terms are required.

Finally, we expand this method to excited state calculations, by employing the folded-spectrum approach, in which the Hamiltonian is replaced by a variance operator. The squared operator has significantly more terms than the underlying Hamiltonian, making this method traditionally more expensive than conventional eigensolvers. However, by sampling the operator we find that we can reduce the cost to that of an equivalent ground state calculation.

We believe the results presented here represent a promising proof of concept for the MC-PQE method and its performance should be investigated further for more complex physical systems. As previously noted, this could be made easier by combining the MC-PQE approach with more efficient measurement techniques^28,103−105^ and compact Ansatz encodings,^95,96^ which should significantly reduce required computational time. The latter would also make it possible to move beyond the UCCSD wave function by including higher-order excitations in the cluster expansion, which is prohibitively expensive in the convential UCC encoding scheme. Higher order UCC expansions would allow for a better treatment of more strongly correlated systems and have shown promising results using conventional QMC.^34^

Another alternative to improve the overall accuracy of the method would be to use adaptive Ansätze^25,35,55,56^ or generalized excitations.^126^ However, the use of repeated operators or of operators that do not correspond to excitations from the reference wave function is challenging in projection approaches, due to the requirement of a one-to-one mapping between parametrized Fermionic operators and configurations in the Hilbert space in the residual equations. Developing alternative projection equations that are applicable in this context would therefore be highly useful in order to expand the range of applicability of projective algorithms, both stochastic and deterministic.

The fundamental reason these stochastic approximations and sampling methods work is that, given a wave function that is a steady-state of some operator, they induce fluctuations about this wave function that may be effectively averaged over. Therefore, their applicability is not limited to the projective quantum eigensolver and we believe they may be helpful in any quantum algorithm which aims to converge to a some well-defined final wave function.

## Supporting Information Available

Additional example MC-QMC trajectories employing Hamiltonian term sampling and shift damping (PDF)
