# Supplementary material for: Fighting Noise with Noise: A Stochastic Projective Quantum Eigensolver
Source: J Chem Theory Comput. 2024 Jul 2;20(14):5964–81. doi: 10.1021/acs.jctc.4c00295 (PMC11270749; doi:10.1021/acs.jctc.4c00295)
Supplement: Supplementary file 1 — ct4c00295_si_001.pdf [file ct4c00295_si_001.pdf]

Supplementary Information for “Fighting noise with noise: a stochastic projective quantum eigensolver”

Maria-Andreea Filip*

Yusuf Hamied Department of Chemistry, University of Cambridge, Cambridge CB2 1EW, UK

E-mail: maf63@cam.ac.uk

Example MC-PQE trajectories.

In this appendix we present the MC-PQE trajectories used to generate the data in Table 4 in the main text, as well as to estimate the influence of shift damping on the overall noise of a simulation.

Fig. S1 shows S and E_{proj} over the course of a MC-PQE simulation in which the residuals are computed deterministically, but the Hamiltonian is sampled, for H_3^+. We note that as the number of terms in the sampled Hamiltonian increases, the deviations of the instantaneous estimators from the true energy decay, as expected. This corresponds to decreasing variance of the overall energy estimators.

Fig. S2 shows the same data for a fully stochastic MC-PQE calculation, with 1000 shots per measurement. Similar trends are observed with increasing number of Hamiltonian groups.

Fig. S3 shows the effect of decreasing the damping factor ζ in

$$S(\beta) = S(\beta - A\delta\beta) - \frac{\zeta}{A\delta\beta}\ln\frac{N_{\text{tot}}(\beta)}{N_{\text{tot}}(\beta - A\delta\beta)}, \tag{1}$$

on the fluctuations of the shift and projected energy estimators. While a $10\times$ decrease in ζ reduces the variance of the shift estimator by approximately a factor of 5, it has no effect on E_{proj}, as discussed in more detail in the main text.

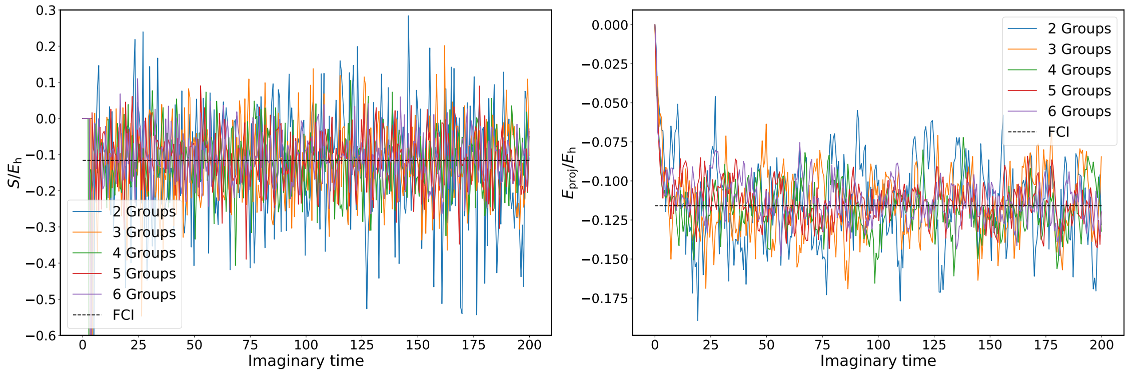

Figure S1: Shift (left) and projected energy (right) as a function of imaginary time obtained by imaginary-time propagation using a stochastically sampled Hamiltonian with only 2-6 Pauli groups selected at each time-step for H_3^+ at $r = 2.0$ Å. The full H_3^+ Hamiltonian has 62 Pauli terms, split here into 25 commuting groups.

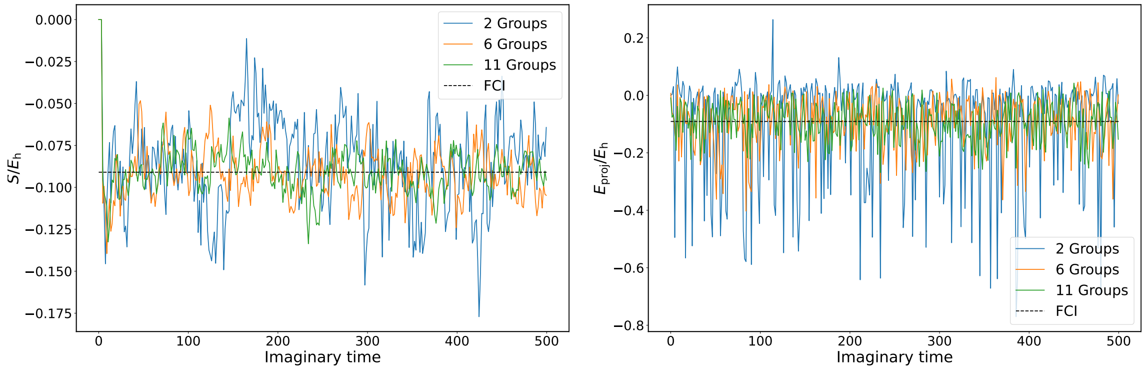

Figure S2: Shift (left) and projected energy (right) as a function of imaginary time obtained by imaginary-time propagation using a stochastically sampled Hamiltonian with only 2-11 Pauli groups selected at each time-step, a stochastically rounded wavefunction and 1000 shots.

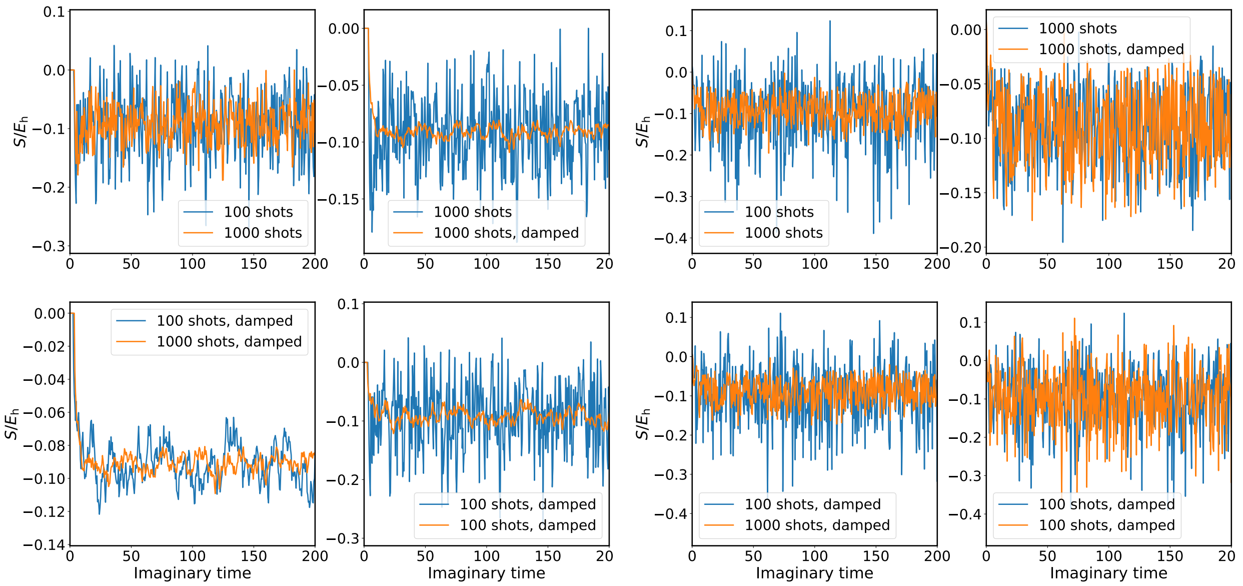

Figure S3: Shift (4 left panels) and E_{proj} (4 right panels) in a simulation of H_3^+ at $r = 1.75$, using 100 or 1000 shots per residual estimation and shift damping parameters $\zeta = 1$ in the undamped case and $\zeta = 0.1$ in the damped case. Shift damping clearly reduces shift fluctuations, while leaving those in the projected energy unchanged.
